# Factors affecting the total occlusion time in eyes with hyperopic anisometropic amblyopia

**DOI:** 10.1186/s12886-023-03206-7

**Published:** 2023-11-20

**Authors:** Keisuke Utamura, Akemi Wakayama, Fumiko Matsumoto, Yukari Shiraishi, Ikumi Narita, Fumi Tanabe, Shunji Kusaka

**Affiliations:** Department of Ophthalmology, Faculty of Medicine, Kindai University, 377-2 Ohnohigashi, Osakasayama, Osaka 589-8511 Japan

**Keywords:** Anisometropic amblyopia, Microtropia, Occlusion therapy, Amblyopia, Total occlusion time

## Abstract

**Background:**

Amblyopia treatment by occluding the healthy eye is known to be effective during a sensitive critical period. This study aims to clarify the factors for the total occlusion time (TOT) required for the amblyopic eye to achieve a normal visual acuity (VA) level of 1.0 (0.0 logMAR equivalent). This could contribute to an efficient treatment plan for eyes with hyperopic anisometropic amblyopia.

**Methods:**

Subjects were 58 patients (26 boys and 32 girls; age range, 3.6-9.2, average, 5.8 ± 1.3 years) with hyperopic anisometropic amblyopia. All the subjects had initially visited and completed occlusion therapy with improved VA of 1.0 or better in the amblyopic eye at Kindai University Hospital between January 2007 and March 2017. Using the subjects’ medical records, we retrospectively investigated five factors for the TOT: the age at treatment, the initial VA of the amblyopic eye, refraction of the amblyopic eye, anisometropic disparity, and the presence of microstrabismus. Patient’s VA improvement at one month after treatment was also evaluated to confirm the effect of the occlusion therapy.

**Results:**

The initial VA of the amblyopic eye ranged from 0.1 to 0.9 (median, 0.4). The TOT ranged from 140 to 1795 (median, 598) hours with an average daily occlusion time of 7 hours. The initial VA of the amblyopic eye and presence of microstrabismus were the significant factors for the TOT (*p* < 0.01). To achieve VA of 1.0 or better, patients with an initial VA of ≤ 0.3 in the amblyopic eye required a longer TOT. Moreover, patients with concomitant microstrabismus required a 1.7-fold longer TOT compared to those without microstrabismus.

**Conclusion:**

Longer daily occlusion hours and early start of the treatment will be necessary for patients with poor initial VA or microstrabismus to complete occlusion therapy within the sensitive critical period.

## Background

Unilateral amblyopia arises from abnormal visual experience when visual information is not properly transmitted to the visual cortex [1]. Its occurrence is usually due to anisometropia or strabismus (including microstrabismus) or a combination of both. When diagnosed early, unilateral amblyopia can be effectively treated by occluding the healthy eye to improve visual acuity (VA) of the amblyopic eye [2–4]. This treatment however is only effective if performed and completed within the critical period with high sensitivity [5–7].

In this study, we also assessed the total occlusion time (TOT) required for patients with various initial VA to achieve a uniform outcome measure (final VA of ≥ 1.0 or 0.0 logMAR equivalent). Because eyes with poor initial VA tend to show more distinct occlusion effect, using a uniform outcome measure for the TOT could avoid the effect of the initial VA differences. To our knowledge, this approach has not been used in studies on amblyopia treatment. Moreover, patient’s VA outcome and cumulative occlusion time at one month after the initiation of occlusion therapy were also investigated to examine their relationship with the final TOT.

For the affected eye to obtain a normal VA level of 1.0 within patient’s critical period, which is the ultimate goal for amblyopia treatment, this study aims to investigate the influential factors for the TOT in patients with anisometropic amblyopia.

## Methods

### Patients

Subjects were 58 patients with unilateral hyperopic anisometropic amblyopia (26 boys and 32 girls; age range, 3.6-9.2 years, average, 5.83 ± 1.33 years). Table 1 shows the subjects’ clinical characteristics.

**Table 1:** Subjects’ clinical characteristics

Refraction value (D)	the healthy eye +2.50 ± 1.30 (0 to 7.75) the affected eye +5.60 ± 1.30 (3.25 to 8.75) anisometropic parallax +3.32 ± 1.22 (1.00 to 6.00)
Visual acuity	the healthy eye median, 1.2 (0.8 to 1.5) the affected eye median, 0.4 (0.1 to 0.9)
Age at treatment (year)	5.8 ± 1.3 (3.6 to 9.2)
Microstrabismus	presence in 22 patients (38%), absence in 36 patients (62%)
Daily occlusion time	5 hours in 21 patients (36%), 8 hours in 37 patients (64%)

Values are mean ± standard deviation

All the subjects had initially visited and completed occlusion therapy with improved final VA of ≥ 1.0 (0.0 logMAR equivalent) in the amblyopic eye at Kindai University Hospital between January 2007 and March 2017. After an optical treatment period of 1 to 2 months, occlusion therapy was initiated when no more VA improvement was observed in the amblyopic eye. In this study, only glasses wear and occlusion of the healthy eye, and no other treatments were performed. Patients of a young age who could not perform VA and fixation tests with a visucope, with previous treatment, spherical equivalent difference between both eyes of < 1.00 D, astigmatism of > 2.00 D, an implementation rate (the number of occlusion hours actually performed ÷ the number of occlusion hours prescribed) of < 80%, or organic diseases (cataract, corneal opacity, ciliary entropion, developmental abnormalities, etc.) were all excluded.

The study protocol was approved by the Ethics Committee of Kindai University and adhered to the tenets of the Declaration of Helsinki. Because all the subjects in this study were under the age of 16, informed consent was obtained from their parents or legal guardians.

### Occlusion period, the TOT, and the influential factors for the TOT

Using the subjects’ medical records, we retrospectively investigated 5 factors for the TOT for the amblyopic eye to achieve VA of 1.0 or better: age at treatment, the initial VA of the amblyopic eye, anisometropic disparity, refraction of the amblyopic eye, and the presence or absence of microstrabismus. The number of occlusion hours performed at home was recorded by the subject every day and used as the daily occlusion time. The duration between the day when the occlusion had been initiated and the day when the amblyopic eye had achieved VA of ≥ 1.0 for the first time was defined as the occlusion period. The TOT (hours) was obtained by multiplying the daily occlusion time (hours) by the occlusion period (days). The presence of microstrabismus was determined by the definition of Lang [8] in patients with eccentric fixation or strabismus of < 10 △.

### Statistical analysis

Multiple regression analysis was used to determine the influential factors for the TOT. The correlation between the TOT and the initial VA of the amblyopic eye was analyzed using Spearman's rank correlation. The influence of the presence of microstrabismus was examined by the Mann-Whitney U test. The difference in the amblyopic eye’s initial VA between the two subgroups was analyzed using Student T test. The amblyopic eye’s VA after 1 month of occlusion therapy was analyzed by the Mann-Whitney U test. A probability level of < 5% was considered statistically significant.

## Results

**Fig. 1:**
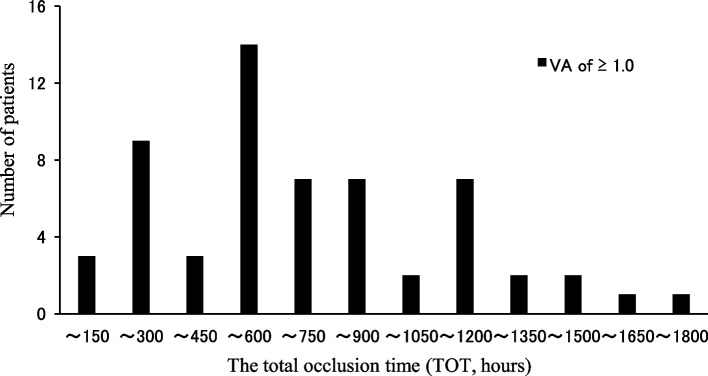
Distribution of the TOT for the amblyopic eye to reach VA of 1.0 or better. Most of the patients (14/58, 24%) required 451- 600 hours in occlusion

The TOT ranged from 140 to 1795 (median, 598) hours. Of 58, 14 (24%) subjects required 451-600 hours (Fig. 1). The average daily occlusion time was 7 hours. Table 2 shows the analysis results for the 5 factors. The presence of microstrabismus and the initial VA of the amblyopic eye showed a significant correlation with the TOT (*p* < 0.01, multiple regression analysis).

**Table 2:** The investigated five factors for the total occlusion time

Factors	Average ± SD	RC	SD	SRC	*P value*
Initial age (year)	5.83 ± 1.33 (3.17-9.17)	28.76	26.99	0.10	0.29
VAA	initial 0.4 (median) (0.1 to 0.9) final 1.0 (median) (0.8 to 1.5)	1127.21	210.37	0.64	<0.01
AP (D)	+3.32 ± 1.22 (1.00 to 6.00)	-42.48	37.54	-0.13	0.26
RVA (D)	+5.60 ± 1.30 (3.25 to 8.75)	8.34	31.49	0.03	0.79
Micro- strabismus	presence in 22 patients (38%) absence in 36 patients (62%)	-241.98	85.67	-0.30	<0.01

*RC* Regression coefficient, *SD* Standard deviation, *SRC* Standard regression coefficient, *VAA* Visual acuity of the amblyopic eye, *AP* Anisometropic parallax, *D* Dioptre; *RVA* Refraction value of the amblyopic eye

### Correlation between the TOT and the presence of microstrabismus

**Fig. 2:**
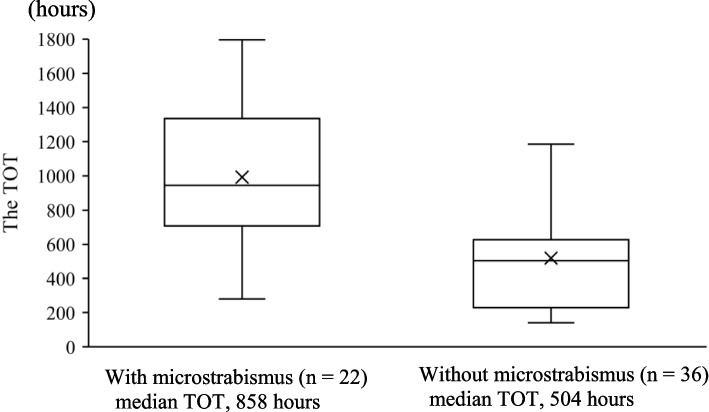
Boxplots showing the TOT for the patients with and without microstrabismus. The top and bottom of the boxes represent the top and bottom quartiles, and the whiskers represent the maximum and minimum values. The TOT for the patients with microstrabismus was significantly longer (*p* < 0.01, Mann-Whitney U test)

The TOT for the subjects with microstrabismus was significantly longer than the TOT for those without microstabismus (*p* < 0.01, Mann-Whitney U test; Fig. 2). Moreover, the subjects with microstrabismus required a 1.7-fold longer TOT than those without microstrabismus to achieve VA of 1.0 in the amblyopic eye.

### Correlation between the TOT and the amblyopic eye’s initial VA

**Fig. 3:**
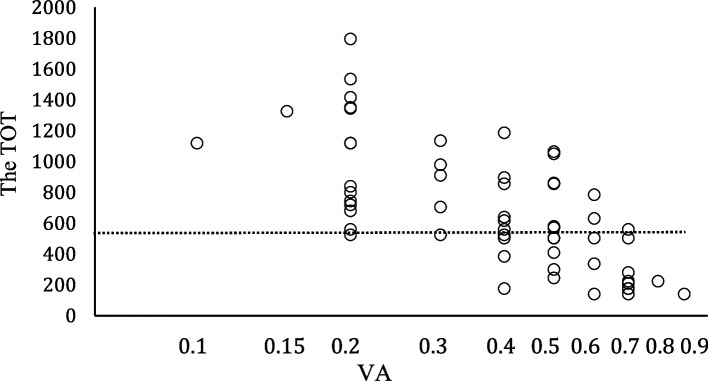
The correlation between the TOT and the initial visual acuity (VA) of the amblyopic eye. A significant negative correlation was observed (rs = -0.72, *p* < 0.01, Spearman's rank correlation coefficient). Of 58, 28 patients required a TOT longer than the median TOT (598 hours) and their median initial VA of the weaker eye was 0.3. The other 30 patients with a TOT less than 598 hours had the median initial VA of 0.5 in the weaker eye

A negative correlation was observed between the TOT and the initial VA of the amblyopic eye (rs = -0.72, *p* < 0.01, Spearman's rank correlation coefficient; Fig. 3). Using the median TOT (598 hours), the correlation was further examined in two subgroups: the subjects with a TOT of < 598 hours (30/58, 52%) and those with a TOT of ≥ 598 hours (28/58, 48%). The amblyopic eye’s initial VA (median) was 0.5 in the subjects with a TOT of < 598 hours and 0.3 in those with a TOT of ≥ 598 hours. A significant difference in the amblyopic eye’s initial VA was observed between these two subgroups (*p* < 0.01, Student T test). The subjects with initial VA worse than 0.3 in the amblyopic eye required a longer TOT.

### The amblyopic eye’s VA after one month of occlusion therapy

At one month after occlusion therapy, the amblyopic eye’s VA** (**median) was 0.9 and the average cumulative occlusion time was 215 hours in the eyes without microstrabismus. Compared to that, the amblyopic eyes with combined microstrabismus had a median VA of 0.5 and an average cumulative occlusion time of 237 hours, showing significant differences from those in eyes without microstrabismus (p < 0.01, Mann-Whitney U test). The eyes without microstrabismus showed a better response to the occlusion therapy

## Discussion

Our results have demonstrated that patients with initial VA of 0.3 or worse in the amblyopic eye or with combined microstrabismus required a longer TOT. Particularly, patients with microstrabismus showed poorer VA improvement in the amblyopic eye one month after the start of occlusion therapy and required a 1.7-time longer TOT than those without microstrabismus. These are important factors for a successful treatment plan in patients with hyperopic anisometropic amblyopia.

The relationship between daily occlusion dose and treatment effect has been studied previously [9–11]. These studies assess the effect based on the VA improvement from baseline in patients with different levels of initial VA [12]. However, patients with different initial VA could contribute to the treatment effect differently even if they have achieved the same level of VA improvement after treatment. For instance, the treatment effects seen in the amblyopic eyes with initial VA of 0.1 and 0.3 (or 1.0 and 0.5 logMAR) and a 2-line VA improvement after occlusion (final VA of 0.5 and 1.0, or 0.3 and 0.0 logMAR) should be considered different although the VA improvements are the same. When measuring the TOT in this study, we therefore used a uniform goal (final VA of 1.0) for all the patients regardless of their amblyopic eyes’ initial VA. This could minimize any possible effect on the assessment results due to their initial VA differences.

Reportedly, 30-50% of anisometropic amblyopia patients have concomitant microstrabismus [13–16]. In patients with anisometropic amblyopia, occlusion therapy is usually initiated after no more VA improvement observed in the amblyopic eye with optical treatment. A previous study however has pointed out the need for occlusion therapy in anisometropic amblyopia patients with combined microstrabismus [17]. Although all the patients in this study (with or without microstrabismus) could achieve a final VA of 1.0 or better in the amblyopic eye, the poor VA improvement at 1 month after occlusion and 1.7-time longer TOT observed in the patients with microstrabismus suggested possible treatment resistance in these microscopic eyes. We suspected that eccentric fixation and strabismus-induced suppression might have caused the longer TOT in these eyes. This also indicated the importance of fixation and suppression tests and the determination of combined microstrabismus before the start of occlusion therapy. It is however not an easy task in clinical practice to determine visual fixation and inhibition before treatment especially in patients of young age. Our results suggest that the VA outcome at one month after occlusion could help distinguish between anisometropic amblyopia patients with and without concomitant microstrabismus.

With this information, patient’s daily occlusion dose can be adjusted accordingly with consideration given to the patient’s required TOT and critical period.

The initial VA of the amblyopic eye was the other significant factor for the TOT. A previous study reported that patients with initial VA of 0.3 to 1.3 logMAR in the amblyopic eye require occlusion for at least 6 hours per day to obtain the maximal VA improvement [10]. The present study also showed that patients with initial VA of 0.3 or worse required a longer TOT than those with initial VA of > 0.3 to achieve a final VA of 1.0 or better. Because the initial VA of the weaker eye indicates the level of the eye’s VA development, it takes a longer treatment period to promote the VA development in amblyopic eyes with worse initial VA. Based on patient’s initial VA of the amblyopic eye, the required TOT can be predicted and the daily occlusion dose can be determined accordingly.

This study has some limitations including the nature of a retrospective study. Because only the patients who could achieve a final VA of ≥ 1.0 in the amblyopic eye and patients with an implementation rate of ≥ 80% for performing occlusion at home were included, the number of subjects in this retrospective study was relatively small. A future prospective study with a large group of patients will be necessary to further assess the relationship between the amblyopic eye’s VA improvement and the TOT.

In conclusion, anisometropic amblyopia patients with initial VA of ≤ 0.3 or concomitant microstrabismus require more total hours in occlusion to obtain a normal VA level of 1.0 or better in the affected eye. Particularly, patients with microstrabismus require a 1.7-time longer TOT than patients without microstrabismus. These results help predict patient’s required TOT and the optimal daily occlusion dose can be determined accordingly. A subsequent evaluation of VA outcomes after one month of occlusion can verify the presence of concomitant microstrabismus and if necessary, the treatment plan can be further modified for better efficiency to complete the treatment within patient’s critical period.

## Data Availability

The datasets used for analysis in this study are available from the corresponding author on reasonable request.

## References

[1] Von Noorden GK. Binocular vision and ocular motility: theory and management of strabismus. CHAPTER 14 Examination of the Patient—IV AMBLYOPIA. 2002. p. 246–50.

[2] Qurrah TI Vision and perception. Riyadh: Obiekan Publishing House; 1991.

[3] Maconachie Gail DE, Gottlob I (2015). The challenges of amblyopia treatment. Biomed J..

[4] Tailor V, Bossi M, Greenwood JA, Noor AD (2016). Childhood amblyopia: current management and new trends. Br Med bull..

[5] Hubel DH, Wiesel TN (1962). Receptive fields, binocular interaction and functional architecture in the cat’s visual cortex. J Physiol..

[6] Hubel DH, Wiesel TN (1963). Shape and arrangement of columns in cat’s striate cortex. J Physiol..

[7] Hensch TK, Quinlan EM (2018). Critical periods in amblyopia. Vis Neurosci..

[8] Lang J (1969). Microtropia. Arch Ophthalmol..

[9] Holmes JM, Kraker RT, Beck RW (2003). A randomized trial of prescribed patching regimens for treatment of severe amblyopia in children. Ophthalmology..

[10] Repka MX, Beck RW, Holmes JM (2003). A randomized trial of patching regimens for treatment of moderate amblyopia in children. Arch Ophthalmol..

[11] Yazdani N, Sadeghi R, Moghaddam HM (2017). Part-time versus full-time occlusion therapy for treatment of amblyopia: a meta-analysis. JCO..

[12] Stewart CE, Stephens DA, Fielder AR, Moseley MJ (2007). Modeling dose-response in amblyopia: toward a child-specific treatment plan. Invest Ophthal & Vis Sci..

[13] Ibironke JO (2011). Microtropia: clinical findings and management for the primary eye care practitioner. Optometry..

[14] Hardman Lea SJ, Snead MP, Loades J, Rubinstein MP (1991). Microtropia versus bifoveal fixation in anisometropic amblyopia. Eye..

[15] Tomac S, Sener EC, Sanac AS (2002). Clinical and sensorial characteristics of microtropia. JJO..

[16] Lang J (1974). Management of microtropia. BJO..

[17] Lysons D, Tapley J (2018). Is microtropia a reliable indicator of the presence of amblyopia in anisometropic patients?. Strabismus..

